# Analysis of Phosphate Transporters in Peritoneal Cells and Tissues and Their Transport Kinetics In Vitro

**DOI:** 10.3390/ijms27083683

**Published:** 2026-04-21

**Authors:** Zhiwei Du, Maria Bartosova Medvid, Iva Marinovic, Sotirios G. Zarogiannis, Claus Peter Schmitt

**Affiliations:** 1 Clinics 1, Pediatric Nephrology, Center for Pediatric and Adolescent Medicine, Medical Faculty Heidelberg, Heidelberg University, 69120 Heidelberg, Germany; zhiwei.du@med.uni-heidelberg.de (Z.D.); maria.bartosovamedvid@med.uni-heidelberg.de (M.B.M.); iva.marinovic@med.uni-heidelberg.de (I.M.); 2 Emergency Department, Clinical Hospital Centre Rijeka, Tome Strižića 3, 51000 Rijeka, Croatia; 3 Department of Physiology, Faculty of Medicine, School of Health Sciences, University of Thessaly, BIOPOLIS, 41500 Larissa, Greece; szarog@med.uth.gr

**Keywords:** phosphate, peritoneal dialysis, Transwell system, mesothelial cells, endothelial cells

## Abstract

Peritoneal dialysis (PD) is limited by insufficient phosphate removal, leading to adverse cardiovascular outcomes in patients with chronic kidney disease. To advance the understanding of the molecular mechanisms of peritoneal phosphate transport, RNAseq data of phosphate transporters in four PD-relevant cell lines were analyzed. The expression and localization of the respective proteins were validated by immunostaining in these cells. The transcriptomics of omental arterioles from children on PD were analyzed. In vitro Transwell models of an immortalized mesothelial cell line (MeT-5A) and human umbilical vein endothelial cells (HUVECs) and respective co-cultures were established, enabling quantification of phosphate transport across mesothelial and endothelial monolayers. Sodium phosphonoformate tribasic hexahydrate (PFA) and Tenapanor were used to inhibit transcellular and paracellular transport pathways. Cell viability and integrity markers were measured over the experimental periods. *SLC20A1* and *SLC20A2* were expressed across all studied cell types, while *SLC34A2* and *SLC34A3* were mesothelial cell-specific. Omental arterioles of children on low-glucose-degradation-product (GDP) PD showed higher *SLC20A1* expression vs. stage 5 chronic kidney disease (CKD5) and healthy controls. Permeability for phosphate was lower across MeT-5A compared with HUVEC monolayers and was not further reduced in co-culture. Inhibitors reduced both transcellular and paracellular transport to 75% in MeT-5A and 65% in co-cultures, while no effects were observed in HUVEC alone, suggesting the mesothelial cell layer as a significant barrier for phosphate transport. Our studies provide first analyses combining findings on molecular phosphate transporters in peritoneal cells and arterioles and introducing a Transwell model for quantitative studies of phosphate kinetics.

## 1. Introduction

Dialysis is a life-maintaining therapy for patients with stage 5 chronic kidney disease (CKD5), also known as end-stage kidney disease (ESKD). Among dialysis modalities, peritoneal dialysis (PD) offers distinct advantages over hemodialysis (HD), including superior preservation of residual kidney function, greater patient autonomy, and lower healthcare costs [1,2,3]. Despite these benefits and its utility in resource-limited settings, PD remains underutilized, accounting for only 11% of dialysis patients globally [4]. A primary factor limiting the wider adoption of PD is the progressive decline in membrane function and insufficient solute clearance, particularly of phosphate [5,6].

Phosphate management is an important challenge in PD [7]. Although phosphate is a small molecule with a molecular weight of only 95 g/mol, its hydration envelope and complex intracellular distribution lead to low clearance rate [8,9,10]. Peritoneal phosphate clearance is approximately 20% lower than clearance of creatinine and 50% lower than urea clearance [5,11,12,13]. Consequently, the prevalence of hyperphosphatemia in children undergoing PD increases with age, from 6% in infants to 81% in adolescents [14]. About 40% of adult PD patients have increased serum phosphate concentrations [15]. This retention is clinically significant, as high phosphate concentrations are independently associated with vascular calcification, cardiovascular morbidity, and mortality [16,17,18]. Current management strategies, such as dietary restrictions and oral phosphate binders, place a heavy burden on patients and often fail to achieve adequate control [19,20].

Developing novel approaches to enhance dialytic phosphate clearance requires a deeper understanding of peritoneal transport mechanisms. While the classical “three-pore model” explains fluid and small-solute removal [21,22], recent evidence supports a multi-layered barrier model involving the microvascular endothelium, interstitial matrix and the mesothelium [23,24]. The mesothelium has been considered an insignificant, passive barrier, but recent evidence suggests regulation of small-solute transport via tight junction proteins, such as claudin-2 [23,24]. However, the specific molecular machinery governing phosphate transport across these peritoneal layers remains uncertain.

Systemic phosphate homeostasis in other tissues, such as kidney or intestine, is well characterized and relies on two complementary pathways: transcellular and paracellular transport [25]. The active transcellular pathway is mediated by sodium–phosphate cotransporters of the *SLC34* and *SLC20* families [26], while the passive paracellular pathway is modulated by the permeability of tight junctions [27,28]. Phosphonocarboxylates are efficacious competitive inhibitors, among which sodium phosphonoformate tribasic hexahydrate (PFA) is a well-studied inhibitor for both *SLC34* and *SLC20* families, i.e., the transcellular phosphate transport [29,30]. Tenapanor inhibits the sodium–hydrogen exchanger 3 (NHE3) and modulates tight junctions, which results in lower paracellular permeability of phosphate and in the intestinal tract in reduced Pi absorption [31,32]. Their potential effects on peritoneal phosphate transport have not yet been explored.

Taken together, there is an essential need for understanding the cellular and molecular mechanisms of peritoneal phosphate transport, to overcome a major limitation of PD, insufficient dialytic phosphate clearance. We therefore studied phosphate transporter expression in four PD relevant cell lines and regulation of these genes in human tissues and established an in vitro phosphate transport model across mesothelial and endothelial monolayers providing functional analyses.

## 2. Results

### 2.1. Phosphate Transporters in Peritoneal Cells and Tissues

Cell lines frequently used in PD-related research projects include human primary mesothelial cells (HPMCs), an immortalized mesothelial cell line derived from a non-cancerous pleural effusion that was subsequently SV-40-immortalized (MeT-5A), and primary human umbilical vein endothelial cells (HUVECs). Human cardiac microvascular endothelial cells (HCMECs) are more difficult to handle and costly and are therefore infrequently used but better reflect the peritoneal transport across capillaries than HUVECs. Ten known phosphate transporters (Table 1) were compared between the four different cell lines for expression and localization. *SLC20* families (Type III), *SLC34* families (Type II) and *SLC17* families (Type I) are sodium-dependent phosphate cotransporters. *SLC53A1* (*XPR1*) is the only known phosphate efflux transporter.

#### 2.1.1. Gene Expression of Phosphate Transporters in Mesothelial and Endothelial Cells

**Figure 1:**
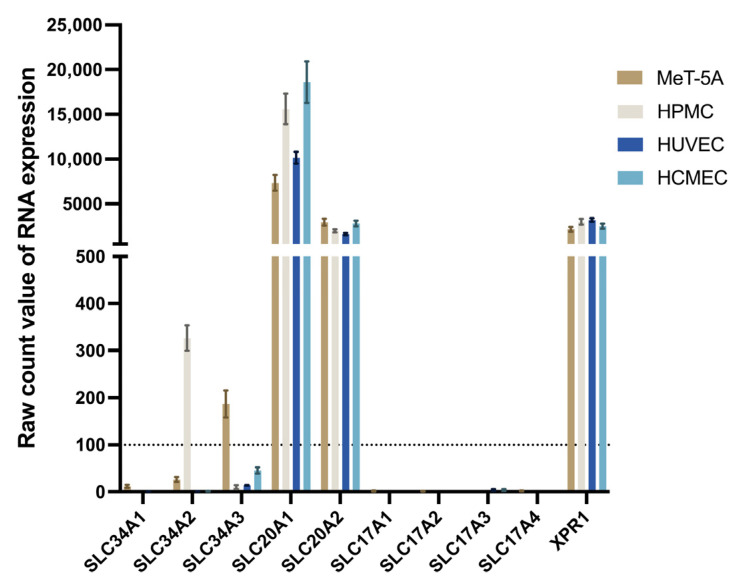
Expression of phosphate transporters in four PD-relevant cell lines. The raw expression count value of phosphate transporters is given on the *y*-axis. A value of 100 was set as the threshold of expression (dashed line, see Section 4). HPMC and MeT-5A are primary and immortalized mesothelial cell lines; HUVEC and HCMEC are primary human umbilical and cardiac microvascular endothelial cells. *SLC20A1* (*PiT-1*), *SLC20A2* (*PiT-2*) and *XPR1* are highly expressed, while *SLC34A2* is HPMC-specific and *SLC34A3* only expressed in MeT-5A. Data are mean ± SD.

RNA-seq data from total RNA with 12,760 unique protein-coding transcripts passing the filtering criteria [33] was analyzed for RNA expression levels of the 10 phosphate transporters. *SLC20A1* (*PiT-1*), *SLC20A2* (*PiT-2*) and *SLC53A1* (*XPR1*) had high expression levels in all four cell types. *SLC34A2* was only expressed in HPMC, while *SLC34A3* was only expressed in MeT-5A, and the expression was lower than expression of *SLC20A1* (*PiT-1*) and *SLC20A2* (*PiT-2*) (Figure 1). Other phosphate transporters had expression level raw count values below the threshold of 100 in all four studied cell lines.

#### 2.1.2. Protein Abundance and Localization of Phosphate Transporters In Vitro

**Figure 2:**
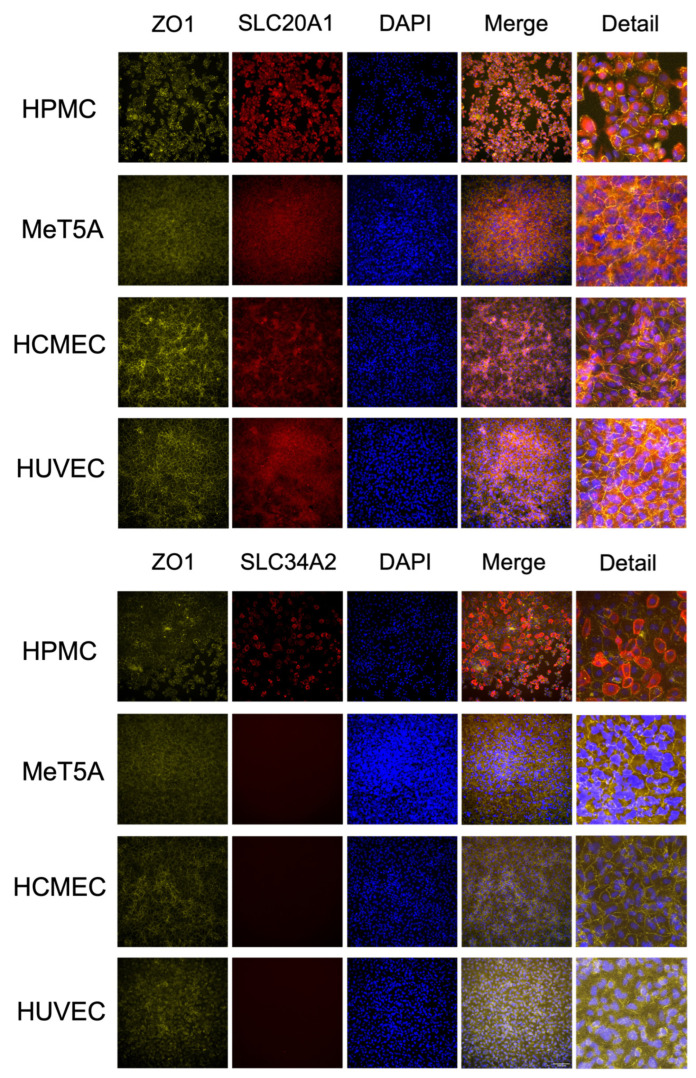
Immunofluorescence staining of SLC20A1 (PiT-1), SLC34A2 and ZO-1 in the two different mesothelial (HPMC, MeT-5A) and endothelial cell lines (HCMEC and HUVEC). The cells were cultured on Transwell filters until confluence. SLC20A1 (PiT-1; red staining, upper panel), SLC34A2 (red staining, lower panel), ZO-1 (green in both panels) and nuclei (DAPI, blue) were stained. SLC20A1 (PiT-1) protein is expressed in both mesothelial and endothelial cell lines while SLC34A2 is HPMC-specific. Scale bar = 100 µm.

To study the protein abundance and localization of two of the highly expressed phosphate transporters, SLC20A1 (PiT-1), and SLC34A2, the four cell lines were seeded in polyester Transwell filters and were cultured for 6 days until confluence and immunofluorescence staining was performed. Filters were cut out from the Transwell filters and mounted on a glass slide; the images are given in Figure 2. SLC20A1 (PiT-1) was expressed in both mesothelial and endothelial cell lines while SLC34A2 was HPMC-specific, which reconfirmed the RNA-seq results. Tight junction protein ZO-1 was co-stained to show the cell junction and cell border and DAPI was used for the nuclei position, showing that SLC20A1 (PiT-1) and SLC34A2 were mostly co-localized at the cell membrane.

#### 2.1.3. Phosphate Transporters in Human Arterioles of Children with Normal Kidney Function, with CKD5 and on PD

**Figure 3:**
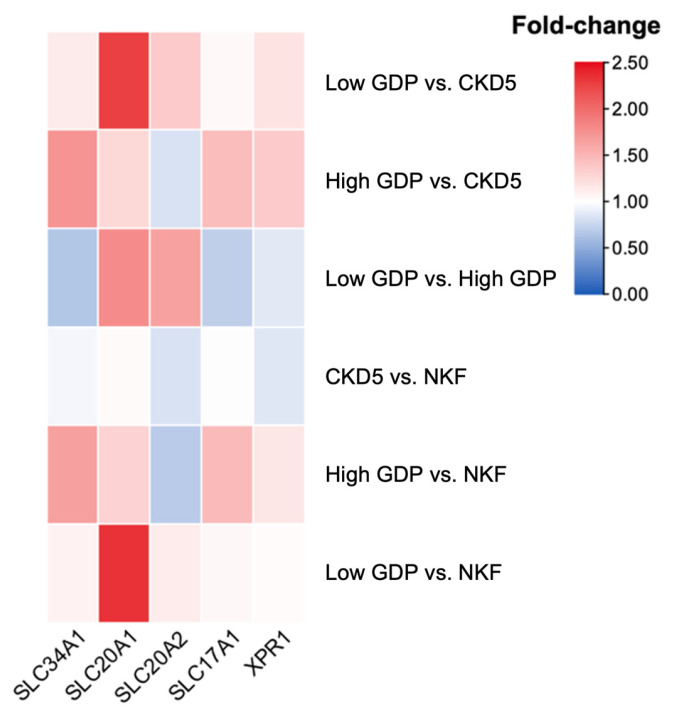
Transcriptional profiles of phosphate transporters. The heatmap represents the fold change in transcripts of five phosphate transporters in microdissected omental arterioles of children with NKF, CKD5 and on PD. Darker red represents higher RNA expression levels. *SLC20A1* (*PiT-1*) expressed is two-fold higher in low-GDP PD patients compared to children with CKD5 and NKF.

Microdissected omental arterioles obtained from children with normal kidney function (NKF), eight with CKD5 and five and six on PD with dialysis fluids containing low or high concentration of GDP, respectively [34], were studied for the expression of phosphate transporters (for cohorts see Table S1) [35]. Five of the 10 phosphate transporters were detected, *SLC34A1*, *SLC20A1* (*PiT-1*), *SLC20A2* (*PiT-2*), *SLC17A1* and *XPR1*. Arteriolar *SLC20A1* (*PiT-1*) RNA was two-fold more abundant in patients treated with low-GDP PD fluid compared to those with CKD5 and NKF (Figure 3).

These in vitro and ex vivo findings in peritoneal cells and tissues laid the molecular basis for the in vitro studies of peritoneal phosphate transport.

### 2.2. Phosphate Transport Kinetics In Vitro

To systematically study phosphate transport across polarized mesothelial and endothelial monolayers in vitro, MeT-5A and HUVEC were either seeded separately or co-cultured on the 0.4 µm PE mesh Transwell filter to mimic the peritoneal membrane structure. A cell medium with different phosphate concentrations was added in the apical part of the Transwell, the concentration of phosphate transported to the basolateral side was repeatedly measured and the apparent permeability coefficient (*P*_app_) for phosphate was calculated. Specific blockers were added to distinguish the transcellular and paracellular transport routes.

#### 2.2.1. Phosphate Transport Across Mesothelial Cell Monolayers

Several experimental set-ups with varying phosphate concentrations and times were studied, based on the following considerations. Phosphate transporters have different affinities for monovalent (H_2_PO_4_^−^) and divalent (HPO_4_^2−^) ions and are pH-dependent [26]. Physiologically, the pH of the human body ranges between 7.35 and 7.45, with the average at 7.40 [36]. To demonstrate the physiological molecular functions of these transporters better in vitro, phosphate buffer with a mixture of NaH_2_PO_4_ and Na_2_HPO_4_ at pH of 7.40 was applied in the system.

**Figure 4:**
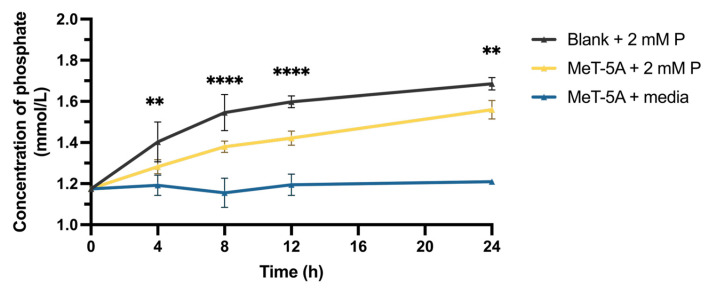
Phosphate transport across polarized MeT-5A mesothelial cell monolayers. Phosphate (P) concentration in the basolateral part of the Transwell over 24 h with 2 mM P added in the apical part. P concentration increased in the basolateral cell media significantly less over 24 h across the confluent, polarized mesothelial cell monolayers than across the cell-free, blank filter. (*n* = 3 independent experiments in duplicates; data are presented as mean ± SD; ** *p* < 0.01, **** *p* < 0.0001, two-way ANOVA with Dunnett’s multiple comparisons).

Considering the prevalent hyperphosphatemia in adults with serum phosphate concentration > 1.46 mmol/L (>4.5 mg/dL) [37], the higher physiological age-dependent serum concentrations in children and the toxicity of increased phosphate concentration, a range of phosphate concentrations were studied in MeT-5A. Cell viability was assessed by MTT (Figure 4) every 4 h for a total of 24 h (Figure S1A); MTT did not demonstrate toxicity in MeT-5A with phosphate concentrations below 8 mM, but transepithelial resistance (TER), a measure of cell monolayer integrity, declined with phosphate concentrations above 2 mM (Figure S1B). Based on this and a series of subsequent pilot experiments with different phosphate concentrations in the apical compartment, phosphate concentrations of 1 and 2 mM were used in the apical side of the Transwell system for the subsequent transport studies.

Adding 2 mM of phosphate in the apical and 1.2 mM (as originally present in M199 medium) in the lower compartment, the concentration of phosphate in the basolateral side of the Transwell with a MeT-5A cell monolayer increased over time, but the increase was significantly less pronounced than in the blank Transwell without MeT-5A cells. The differences in concentrations between blank and MeT-5A cell monolayers were most pronounced at 8 h and 12 h. In the Transwell containing only MeT-5A cells in media without added phosphate, the phosphate concentrations remained unchanged (Figure 4).

Based on these findings, subsequent phosphate transport studies in MeT-5A were performed with 1 mM or 2 mM phosphate-containing cell culture medium in the apical compartment and 0.1 mM phosphate-containing culture medium in the basolateral compartment. Samples from both compartments were taken at baseline and at 4, 8 and 12 h time points. The phosphate transport is presented as the percentage of the expected equilibrium which has been achieved after the given time point and the apparent permeability coefficient (*P*_app_), with higher values representing higher transport due to a more permeable cellular barrier and lower values representing less transport because of tighter cellular barrier.

**Figure 5:**
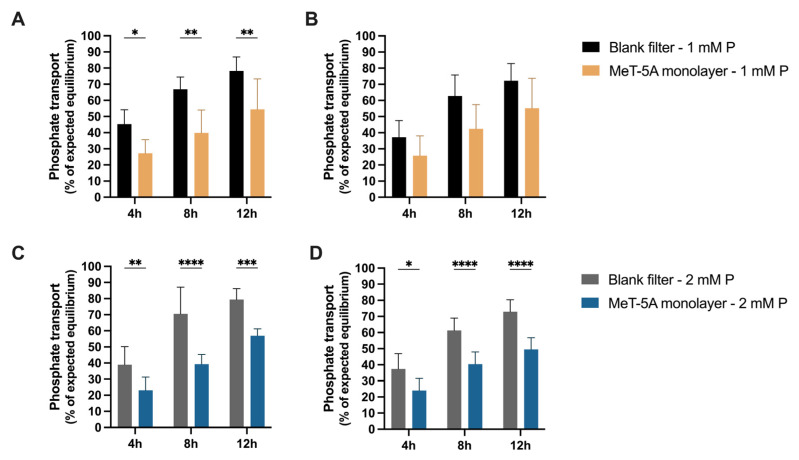
Phosphate transport kinetics across polarized MeT-5A cell monolayers. With 1 mM (**A**) and 2 mM P (phosphate) (**C**) added to the apical side, and 0.1 mM phosphate-containing medium in the basolateral compartment, phosphate transport to the lower compartment (shown as percentage of the expected equilibrium) was significantly lower across MeT-5A monolayers than across cell-free, blank filters. Similar findings were obtained for the apical phosphate concentration kinetics (**B**,**D**). (All data are mean ± SD. *n* = 3 experiments in duplicates; * *p* < 0.05, ** *p* < 0.01, *** *p* < 0.001, **** *p* < 0.0001, two-way ANOVA with Šídák’s multiple comparisons).

With 1 mM phosphate added to the apical compartment in the MeT-5A cell line, the phosphate concentration in the basolateral compartment increased slowly over time and achieved 54.4 ± 18.9% of the expected equilibrium after 12 h. The transport across the MeT-5A cell monolayer was again significantly slower than across cell-free blank filters, in which the phosphate concentration in the basolateral compartment reached 78.1 ± 8.7% of the equilibrium after 12 h (*p* = 0.0068 vs. MeT-5A) (Figure 5A). Comparable results were obtained for the apical kinetics. Within 12 h, 55.2 ± 18.5% of equilibrium was reached in MeT-5A compared to 72.2 ± 10.6% of the equilibrium in blank filters (Figure 5B). Similar relative phosphate transport rates were obtained with 2 mM phosphate in the apical compartment as with 1 mM phosphate (Figure 5C,D), with differences being highly significant at each time point of the experiment. Absolute transport rates were higher with 2 mM than 1 Mm phosphate.

#### 2.2.2. Phosphate Transport Across Endothelial Cell Monolayers

The effect of increasing concentrations of phosphate on HUVEC viability was measured with MTT (Figure S1C). Within 12 h of incubation, reduced cell viability was only observed with the highest phosphate concentration of 8 mM. Lower phosphate concentrations did not affect HUVEC viability, but even increased the reduction of MTT to Formazan, which is a correlate of metabolic cell activity.

**Figure 6:**
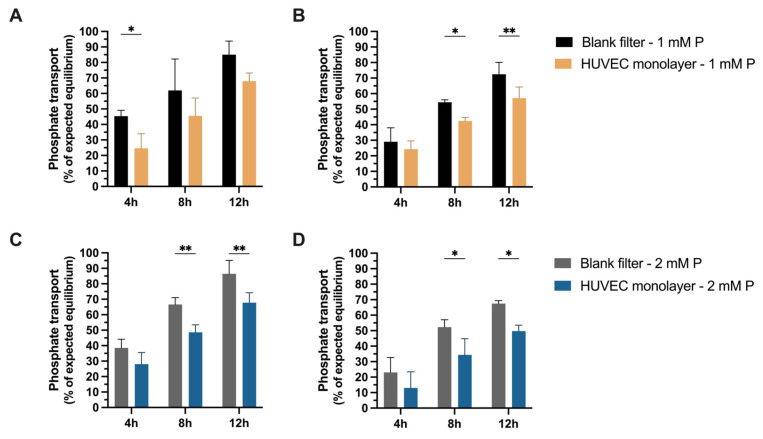
Phosphate transport across polarized HUVEC monolayers. The increase in phosphate (P) concentrations in the basolateral side of the Transwell over time was quantified with 1 mM (**A**) and 2 mM of P added in the apical compartment (**C**). The respective reduction in P concentrations in the apical compartment is given in (**B**,**D**). (Data are mean ± SD. *n* = 3 experiments in duplicates; * *p* < 0.05, ** *p* < 0.01, Two-way ANOVA with Šídák’s or Dunnett’s multiple-comparisons test).

As for MeT-5A phosphate transport was measured across confluent, polarized HUVEC monolayers in Transwells. Within 12 h phosphate transport from the apical into the basolateral side reached about 70% of the expected equilibrium with 1 mM (Figure 6B) and 2 mM of phosphate added to the apical side (Figure 6D), respectively. The decrease in the phosphate concentration in the apical side corresponded to the increase in the basolateral side (Figure 6B,D). Phosphate transport across cell-free Transwell filters was consistently higher than in filters with HUVEC (Figure 6A–D).

#### 2.2.3. Co-Culture Studies of Phosphate Transport

To better mimic the peritoneal membrane during PD, a co-culture system with both endothelial and mesothelial cells was used. Four different models (MeT-5A on the apical side/MeT-5A on the basolateral side/HUVEC on the apical side/co-culture of HUVEC on the apical and MeT-5A on the basolateral side) were compared. For this, 1 mM or 2 mM phosphate was added into the apical side. Transepithelial resistance (TER) was measured before and after the 8 h treatment (Figure S1D). Co-culture models showed the highest TER while HUVEC alone showed the lowest, reflecting the higher ionic conductance of HUVEC monolayers. The TER of only MeT-5A monolayers was between the other two cultures. Addition of phosphate to the apical compartment did not change TER systematically.

**Figure 7:**
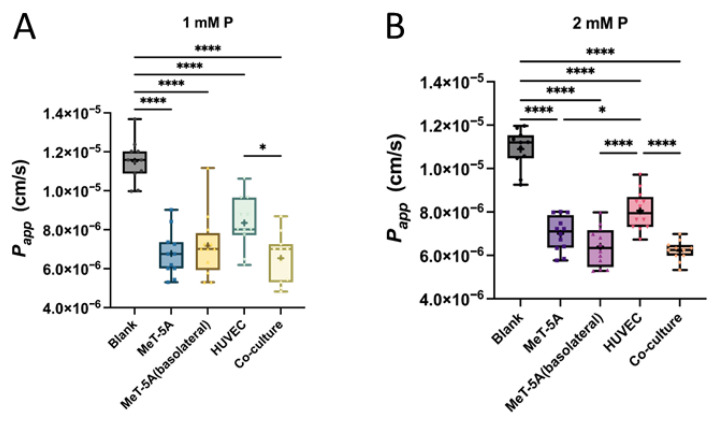
Apparent permeability coefficient (*P*_app_) of phosphate across confluent cell monolayers after 8 h. *P*_app_ is given for MeT-5A monolayers on the apical side, MeT-5A on the basolateral side, HUVEC on the apical side and the co-culture of HUVEC and MeT-5A and blank (filter without cells). Either 1 mM (**A**) or 2 mM (**B**) of phosphate was added to the apical side. Data are given as minimal to maximal value; + represents mean value (*n* = 5 independent experiments in duplicates; * *p* < 0.05, **** *p* < 0.0001, one-way ANOVA with Tukey’s multiple-comparisons test).

*P*_app_ of phosphate across all four cell models was calculated after 8 h. *P*_app_ of phosphate (Figure 7A,B) across all cell monolayers was significantly lower than across blank Transwells. *P*_app_ was highest across HUVEC monolayers. No differences in *P*_app_ were observed when MeT-5A was seeded on the apical or basolateral side of the Transwell and when MeT-5A was seeded on the basolateral side in co-culture with HUVEC. This again demonstrates the low barrier function of the endothelial cells. These findings were consistent with both apical phosphate concentrations.

#### 2.2.4. Transcellular and Paracellular Phosphate Transport

To distinguish transcellular and paracellular transport pathways of phosphate across the different cell monolayers, two different blockers were applied. PFA is an inhibitor of transcellular phosphate transporters. Tenapanor is an NHE3 blocker; it indirectly inhibits paracellular phosphate transport. Either 1 mM PFA in medium or mixture of 1 mM PFA with 1 µM Tenapanor was used and added to the apical and the basolateral side of the Transwell for 1 h pre-incubation. Then these media were removed and media with 1 mM and 2 mM of phosphate in the apical compartment together with PFA or with Tenapanor were added.

##### Transcellular and Paracellular Transport Across Mesothelial Cells

To study the integrity of MeT-5A exposed to 1 and 2 mM phosphate and the inhibitors Tenapanor and PFA grown in the Transwell, cell damage marker LDH, cell viability marker MTT and TER were measured over 12 h (Figure S2A,B). No signs of cell damage and reduced cell monolayer integrity were detected. Immunofluorescence staining of the MeT-5A protein ZO-1 did not suggest tight junction dysregulation (Figure S2C).

**Figure 8:**
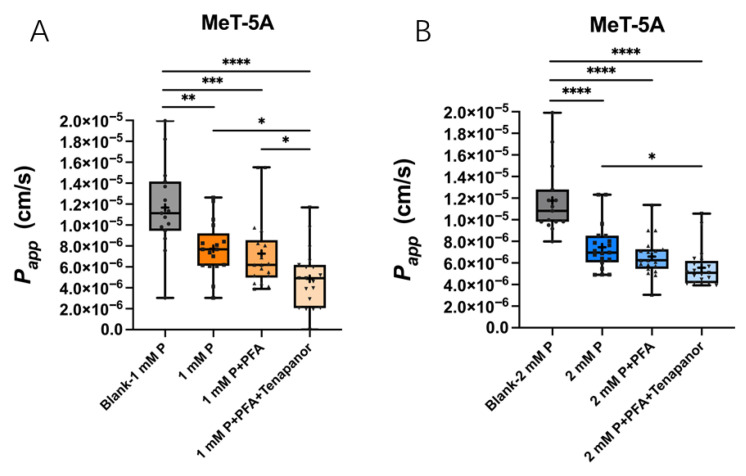
Apparent permeability coefficient (*P*_app_) for phosphate across MeT-5A monolayers with inhibitors of trans- (PFA) and paracellular phosphate transport routes (Tenapanor). *P*_app_ of phosphate decreased by the MeT-5A monolayers compared to blank Transwells and *P*_app_ of phosphate decreased by the combination of PFA and Tenapanor when either 1 mM P (**A**) or 2 mM P (**B**) was added in the apical side of the MeT-5A in Transwells during 12 h. Data are minimal to maximal value; + represents mean value; *n* = 3 independent experiments in duplicates. * *p* < 0.05, ** *p* < 0.01, *** *p* < 0.001, **** *p* < 0.0001, one-way ANOVA with Tukey’s multiple-comparisons test was used.

The combination of PFA and Tenapanor reduced the *P*_app_ of phosphate across MeT-5A compared to 1 mM phosphate without inhibitors added in the apical side (4.86 ± 2.87 × 10^6^ vs. 7.74 ± 2.56 × 10^6^ cm/s, *p* = 0.0158) and with PFA alone (7.26 ± 3.46 × 10^6^ cm/s, *p* = 0.0472) (Figure 8A). These two inhibitors together also reduced the *P*_app_ of phosphate when 2 mM phosphate was added (5.56 ± 1.81 × 10^6^ vs. 7.45 ± 2.16 × 10^6^ cm/s, *p* = 0.0111) (Figure 8B).

When 2 mM phosphate was added in the apical side, a significant inhibitory effect was only achieved with the combination of both 1 mM PFA and 1 µM Tenapanor (Figure 8B).

##### Transcellular and Paracellular Phosphate Transport Across Endothelial Cells

**Figure 9:**
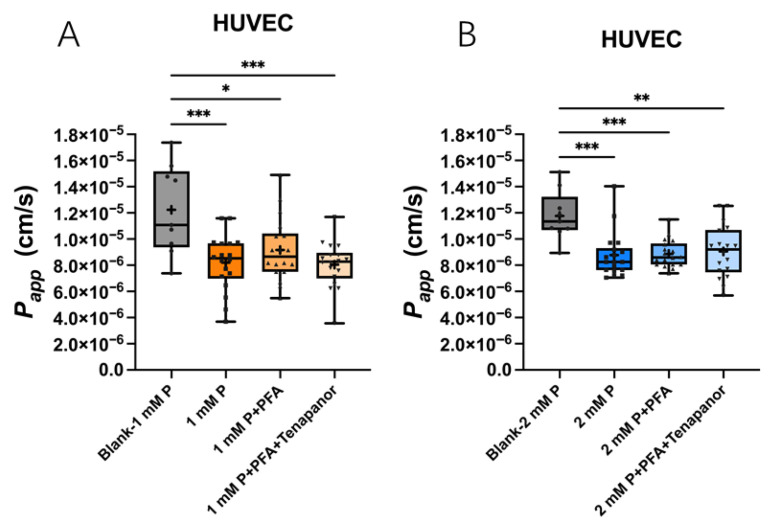
Apparent permeability coefficient (*P*_app_) across HUVEC monolayers with inhibitors of trans- and paracellular phosphate transport. The *P*_app_ of phosphate (P) decreased by the HUVEC monolayers compared to blank Transwells when either 1 mM P (**A**) or 2 mM P (**B**) was added in the apical side of Transwells during 12 h. PFA or the combination of both inhibitors (PFA and Tenapanor) did not change the *P*_app_. *n* = 6 independent experiments in duplicates. Data are minimal to maximal value; + represents mean value (* *p* < 0.05, ** *p* < 0.01, *** *p* < 0.001, one-way ANOVA with Tukey’s multiple comparisons).

In analogy to MeT-5A, HUVEC monolayers were studied in Transwells regarding cell viability (Figure S3A), TER (Figure S3B) and LDH release in the presence of 1 mM and 2 mM phosphate in the apical compartment and 1 mM PFA and 1 µM Tenapanor inhibitors in both compartments, respectively. The treatments did not alter cell viability or TER. All LDH concentrations were below the detection threshold (14 U/L). Permeability for phosphate was lower across HUVEC monolayers than across blank Transwells. Addition of neither PFA nor the combination of both inhibitors, PFA and Tenapanor significantly reduced the *P*_app_ of 1 (Figure 9A) and 2 mM (Figure 9B) phosphate across the HUVEC monolayers.

##### Transcellular and Paracellular Phosphate Transport Across MeT-5A and HUVEC Co-Cultures

**Figure 10:**
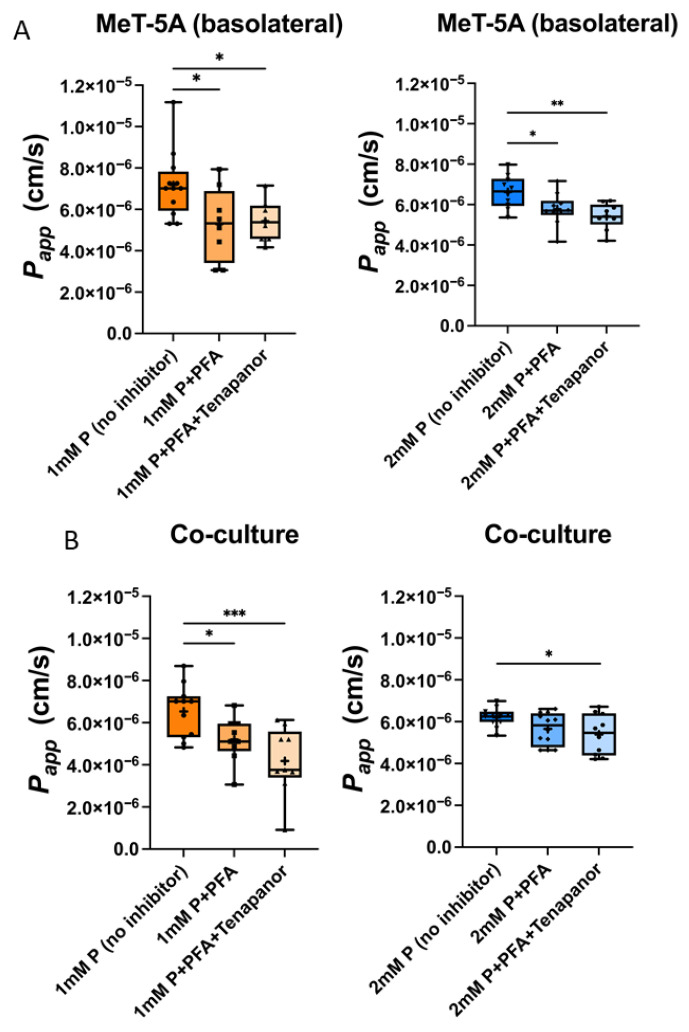
Apparent permeability coefficient (*P*_app_) across HUVEC and MeT-5A co-cultures and inhibitors of trans- (PFA) and paracellular phosphate transport (Tenapanor). (**A**) *P*_app_ of phosphate over 8 h across MeT-5A cultured on the basolateral side of the Transwell. PFA and the combination of PFA with Tenapanor significantly reduced *P*_app_. (**B**) *P*_app_ of phosphate over 8 h across the co-culture of confluent HUVEC and MeT-5A. PFA significantly reduced *P*_app_ when 1 mM P was added in the apical compartment and the combination of PFA with Tenapanor reduced *P*_app_ significantly when either 1 or 2 mM P was added. (All data are minimal to maximal value, + represents mean value, *n* = 6 in duplicates; * *p* < 0.05, ** *p* < 0.01, *** *p* < 0.001, one-way ANOVA with Dunnett’s multiple comparisons and Kruskal–Wallis test with Dunn’s multiple comparisons).

Next, the inhibitors were added at the same concentrations to the co-culture of MeT-5A and HUVEC in the Transwell system with endothelial cells cultured on the apical side, where phosphate was also added, and mesothelial cells on the basolateral side. This mimics the PD conditions in vivo. Over the 8 h treatment, 1 and 2 mM of phosphate and addition of the P-transport inhibitors did not impact TER (Figure S3C). *P*_app_ of phosphate significantly reduced by additional PFA compared to only 1 mM phosphate without inhibitors (5.29 ± 1.77 × 10^−6^ vs. 7.18 ± 1.61 × 10^−6^ cm/s, *p* = 0.0429) and 2 mM phosphate (5.77 × 10^−6^ ± 7.96 × 10^−7^ vs. 6.62 × 10^−6^ ± 8.16 × 10^−7^ cm/s, *p* = 0.0487) across MeT-5A seeded on the basolateral side over 8 h (Figure 10A). The combination of PFA and Tenapanor reduced *P*_app_ of phosphate to 5.43 ± 1.00 × 10^−6^ cm/s (*p* = 0.0359) and 5.42 × 10^−6^ ± 6.46 × 10^−7^ cm/s (*p* = 0.0056) with 1 and 2 mM phosphate separately. Across the co-cultured HUVEC and MeT-5A, PFA reduced the *P*_app_ of phosphate to 5.21 ± 1.07 × 10^−6^ compared to 1 mM phosphate (6.54 ± 1.26 × 10^−6^ cm/s, *p* = 0.0361) and additional Tenapanor caused decrease to 4.18 ± 1.64 × 10^−6^ cm/s (*p* = 0.0006) (Figure 10B) over 8 h. When 2 mM phosphate was added, PFA with Tenapanor reduced *P*_app_ of phosphate to 5.39 × 10^−6^ ± 9.66 × 10^−7^ cm/s compared to phosphate only (6.21 × 10^−6^ ± 4.60 × 10^−7^ cm/s) (*p* = 0.0335).

## 3. Discussion

PD is an essential kidney replacement therapy, particularly for pediatric patients and individuals with limited access to hemodialysis [38]. However, its long-term effectiveness is constrained by inadequate solute clearance, most notably phosphate, which contributes substantially to cardiovascular morbidity and mortality in PD patients [10]. Despite its clinical importance, the molecular mechanisms of phosphate transport across the peritoneal membrane have been incompletely understood [39]. Sodium–phosphate cotransporters (*SLC20*, *SLC34* and *SLC17* family) have been described in various tissues such as intestinal and renal epithelial cells, but their characteristics and contributions in peritoneal cells were unclear [26,40,41]. Understanding phosphate transporter expression and function in mesothelial and endothelial cells is important for developing therapeutic approaches to improve phosphate clearance with PD.

In this study, we demonstrate that phosphate transport across the peritoneal barrier is a regulated and cell-type-specific process involving both mesothelial and endothelial cell monolayers. We observed high expression of Type III sodium–phosphate cotransporters *SLC20A1* (*PiT-1*) and *SLC20A2* (*PiT-2*) and the phosphate efflux transporter *XPR1* across the four studied cell types (MeT-5A, HUVEC, HPMC, HCMEC). Expression of the Type II transporter *SLC34A2* was HPMC-specific and *SLC34A3* expression only in MeT-5A. Both are sodium-dependent phosphate cotransporters and both are expressed only at low levels in the two cell lines; still, this cell-type-specific expression may impact the phosphate transport studies across mesothelial cells. The transcriptomics analysis of microdissected omental peritoneal arterioles from children on PD confirmed the expression of multiple phosphate transporters, including *SLC20A1* (*PiT-1*), *SLC20A2* (*PiT-2*), *XPR1*, *SLC34A1* and *SLC17A1*. Among these genes, *SLC20A1* (*PiT-1*) RNA expression was two-fold more abundant in arterioles from children treated with low-GDP PD fluid compared to non-dialyzed CKD5 patients and children with normal kidney function. This suggests an adaptive response of the vascular system to the stress of uremia, and high systemic phosphate concentrations. Interestingly, *SLC20A1* (*PiT-1*) is closely related to vascular smooth muscle cell calcification [42]. Whether the high *SLC20A1* (*PiT-1*) RNA expression observed in the arterioles from patients on low-GDP PD contributes to vascular remodeling and calcification requires mechanistic studies [34,43,44]. We previously demonstrated by immunohistochemistry the SLC20A1 (PiT-1) abundance in both mesothelial and endothelial cells of the parietal peritoneum in 93 individuals across age groups and in children with CKD5 and on PD [23]. These findings extend knowledge of phosphate transport in PD-relevant cells and suggest that the peritoneum is an active regulator of phosphate transport.

To study the function of these transporters, an in vitro Transwell system allowing direct quantification of phosphate transport kinetics was developed. The Transwell system provides a controlled two-chamber environment that mimics the separation between dialysate and bloodstream compartments as well as the cellular barriers of the peritoneal membrane. The cells grown on the Transwell insert are polarized, enabling directional measurements of solute transport [45].

Using this quantitative Transwell model, baseline phosphate transport rates across MeT-5A were slow. The concentration of phosphate in the basolateral compartment reached the level of blank filters only after 48 h. Only 60% of phosphate transport equilibrium was achieved after 12 h, with the same relative transport results for 1 mM or 2 mM phosphate added to the apical compartment. Also measuring the phosphate appearance in the basolateral and the disappearance in the apical compartment yielded consistent results. This largely excludes technical issues with phosphate measurement with the different cell media compositions and system inherent bias, e.g., due to adsorption to the Transwell membrane or major intra- or extracellular phosphate shifts with time. Phosphate binds to many molecules; considering the bound phosphate, intracellular phosphate concentrations are 10- to 100-fold higher than extracellular concentrations. Our functional transport data are consistent with the persistently low phosphate clearance observed clinically in PD [5,11,46]. We calculated apparent permeabilities for phosphate (*P*_app_), showing that relative to the MeT-5A mesothelial cell monolayer, the endothelial cells (HUVEC) provide comparatively little barrier to phosphate transport. This finding is consistent with the previous findings of our group; endothelial cells are leakier and have a higher permeability compared to mesothelial cells [24].

Pharmacological inhibition experiments indicate that both transcellular and paracellular pathways contribute to phosphate transport across the mesothelium. Partial inhibition by sodium phosphonoformate provides a role for sodium-dependent phosphate cotransporters, while additional suppression by Tenapanor observed in the co-culture system suggests tight junction-mediated paracellular diffusion. This indicates that phosphate clearance across the peritoneal cell barriers is a complex, regulated process involving both transcellular and paracellular routes, similar to what has been described in other epithelial barriers [27,47]. Of note, further studies such as two-path impedance spectroscopy analyses are required to confirm these conclusions. The observed incomplete inhibition suggests the involvement of additional transport mechanisms or compensatory pathways, underscoring the complexity of peritoneal phosphate transport. It is also conceivable that the *K_i_* value of competitive inhibitor PFA is not high enough to achieve a complete blockade, since no successful inhibitors of Pi transport function have been described for the Type III transporters [29].

In PD patients, individual peritoneal phosphate clearance correlates with dwell time and volume, PDF type, serum phosphate concentrations and the individual peritoneal transporter status assessed during the peritoneal equilibration test [11,48,49]. Genome-wide association studies assessed correlations of common genetic variants with peritoneal transfer rates of creatinine and UF [50,51], but not regarding phosphate clearance. In view of the active and passive phosphate transport pathways, as suggested in this paper, a genetic impact is likely, and should be modified by PD fluid type, PD duration and PDF composition. Beyond optimized PD, dietary phosphate restriction and oral phosphate binder intake, which have major adherence issues [52,53,54], novel approaches should be envisaged, specifically enhancing trans- and paracellular phosphate transport activity.

Several limitations should be acknowledged. The in vitro Transwell system represents a simplified model that lacks key in vivo determinants of peritoneal transport, including blood flow, lymphatic drainage, immune interactions, extracellular matrix composition, and mechanical forces [55,56]. The Transwell filter itself constitutes a diffusion barrier, limiting a more precise quantification of cellular barrier contributions. Moreover, the short duration of in vitro experiments does not recapitulate long-term adaptive changes that occur during chronic PD. Ex vivo analyses were limited by the sample size and the cross-sectional design. While HUVECs and MeT-5A cells provided complementary insights, they cannot fully capture the difference in peritoneal endothelial and mesothelial cells in vivo. We used phosphate concentrations of 1 and 2 mmol/L for transport studies, while serum phosphate concentrations may even reach 3 mmol/L, which are detrimental and associated with a poor long-term patient prognosis [10,57]. In line with this, in our cell model, 3 mM phosphate concentrations reduced cell monolayer integrity. Pharmacological inhibition achieved only partial suppression of phosphate transport, likely reflecting limited inhibitor specificity, compensatory activation of alternative pathways, or the existence of yet unidentified transport mechanisms [29]. In addition, low-phosphate culture conditions may have influenced cellular metabolic state and transport kinetics [58,59].

In conclusion, our ex vivo and in vitro studies provide molecular and functional insights into mechanisms which should be relevant to phosphate transport in PD. Our in vitro findings demonstrate that phosphate clearance across the cell monolayers present in the peritoneal membrane is a regulated process involving both transcellular and paracellular pathways, with the mesothelium acting as the principal barrier. The established Transwell model enables quantitative, cell-type-specific analysis of phosphate transport and offers a platform for preclinical testing of therapeutic strategies. Future studies should focus on further functional in vitro and in vivo validation of cellular and peritoneal phosphate transport mechanisms, respectively, and should be amended by longitudinal analysis of transporter expression and function during chronic PD. Targeting peritoneal cellular phosphate transport is a promising therapeutic approach to enhance dialytic phosphate clearance and mitigate hyperphosphatemia, i.e., to address a major unmet need in PD therapy.

## 4. Materials and Methods

### 4.1. Cell Culture

Human umbilical vein endothelial cells (HUVECs; PromoCell, Heidelberg, Germany) were cultured in endothelial cell growth medium with Supplement Mix (PromoCell) and 1% penicillin/streptomycin (P/S; Thermo Fisher Scientific, Waltham, MA, USA). Immortalized human mesothelial cells (MeT-5A; LGC Standards, Wesel, Germany) were maintained in Medium 199 (Thermo Fisher Scientific, Waltham, MA, USA) supplemented with 10% fetal bovine serum (FBS; Thermo Fisher Scientific, Waltham, MA, USA) and 1% P/S. HUVECs were used up to passage 6 and MeT-5A cells up to passage 15.

Primary human peritoneal mesothelial cells (HPMCs) were isolated from omental tissue obtained during abdominal surgery from patients with non-kidney failure (NKF), following approval by the Ethics Committee of Heidelberg University (S-501/2018) and written informed consent. Tissue was washed, enzymatically digested with trypsin-EDTA, subjected to red blood cell lysis, and cultured in M199 supplemented with 10% FBS, insulin, transferrin, hydrocortisone, L-glutamine, and P/S. Cells were identified by characteristic cobblestone morphology and used within three passages.

### 4.2. In Vitro Phosphate Transport Models

**Figure 11:**
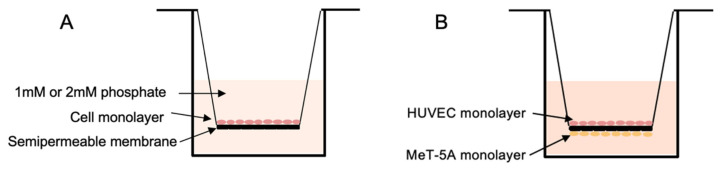
Monolayer and co-culture Transwell system of mesothelial and endothelial cells. (**A**) Cell monolayer in the Transwell insert, with cells on the filter with 200 µL cell culture medium in the apical side and 1000 µL medium in the basolateral side. A cell medium with a modified phosphate concentration (1 mM or 2 mM) was added in the apical part when performing the transport experiments. (**B**) Co-culture system with HUVEC seeded on the apical side of the Transwell membrane and MeT-5A on the basolateral side of the mesh.

Polarized monolayers of mesothelial or endothelial cells were established by seeding 5 × 10^4^ cells onto 0.4 µm pore size polyester Transwell inserts (24-well; SARSTEDT AG & Co. KG, Nümbrecht, Germany). Apical and basolateral compartments contained 200 µL and 1000 µL medium, respectively (Figure 11A). For co-culture experiments, MeT-5A cells were seeded on the underside of inverted inserts, followed by HUVEC seeding on the upper surface after cell attachment (Figure 11B).

Transepithelial electrical resistance (TER) was measured daily using an EVOM volt-ohm meter (World Precision Instruments, Sarasota, FL, USA)until stable monolayers were achieved (4–6 days). TER values were corrected for blank filters and normalized to surface area (0.33 cm^2^).

#### 4.2.1. Phosphate Transport Assays

After monolayer maturation, phosphate transport was initiated using phosphate-free endothelial medium (PromoCell, Heidelberg, Germany) supplemented with 1 or 2 mM phosphate prepared from sterile Na_2_HPO_4_/NaH_2_PO_4_ stock solutions (pH 7.4). Phosphate-containing medium was added apically, with phosphate-free medium added basolaterally. Samples were collected from both compartments at 4, 8, and 12 h. Phosphate and lactate dehydrogenase (LDH) concentrations were quantified by the Central Laboratory of Heidelberg University Hospital. TER was measured during the period.

Ideally, the equilibrium could be achieved regardless of the consumption of phosphate by cells and could be calculated from the initial phosphate concentration. Considering the different volumes of apical side and basolateral side, the expected equilibrium calculation follows the following formula:

*Expected equilibrium* = (*Initial apical phosphate concentration* × 200 + *Initial basolateral phosphate concentration* × 1000)/1200

The expected phosphate transport was defined as the delta of phosphate concentration to the expected equilibrium:

*Apical side expected phosphate transport* = *Initial apical concentration* − *Expected equilibrium*

*Basolateral side expected phosphate transport* = *Expected equilibrium* − *Initial basolateral concentration*

The phosphate transport was defined as the percentage of actual delta of phosphate concentration compared to the expected phosphate transport to the expected equilibrium:

*Phosphate transport in apical side* = (*Initial apical concentration* − *Sample concentration from apical side*)/*Apical side expected phosphate transport* × 100%

*Phosphate transport in basolateral side* = (*Sample concentration from basolateral side* − *Initial basolateral concentration*)/*Basolateral side expected phosphate transport* × 100%

The apparent permeability coefficient (*P*_app_) was calculated by the equation

$$P_{app}=\frac{dQ/dt}{A\times C_{0}}$$

where A is the membrane surface area, C_0_ is the initial donor concentration in the apical compartment, and dQ/dt is the rate of mass appearing in the receiver (basolateral) side.

#### 4.2.2. Paracellular and Transcellular Transport Inhibition

To differentiate transport pathways, cells were pretreated for 1 h with phosphonoformic acid (PFA, 1 mM; Sigma-Aldrich, St. Louis, MO, USA) to inhibit transcellular phosphate transport alone or together with Tenapanor (1 µM; MedChemExpress, Monmouth Junction, NJ, USA) to also inhibit paracellular transport. Phosphate transport assays were then conducted in the continued presence of inhibitors.

### 4.3. Patient Cohorts and Biobank Samples

Peritoneal and omental tissues, blood, and clinical data were obtained from the International Pediatric Peritoneal Biobank (IPPB; NCT01893710) following informed consent and ethical approval (S-493/2018). Cohorts included children with normal kidney function (controls), CKD5, and on PD treated with low- or high-GDP fluids. Patient selection criteria and clinical characteristics are summarized in Table S1.

### 4.4. RNA Sequencing Analysis and Transcriptomics

Transcriptomes of microdissected omental arterioles from children with normal kidney function, with chronic kidney disease and on peritoneal dialysis were generated by our group [35]. These data sets were analyzed for all known phosphate transporters and compared between groups. Our previous RNA-seq data on PD-relevant cells, HPMCs, human cardiac microvascular endothelial cells (HCMECs), human umbilical vein endothelial cells (HUVECs) and immortalized mesothelial cell line (MeT-5A) [33], were assessed for specific phosphate transporter expressions. The raw expression count values of 10 phosphate transporters in these four cell types were analyzed and presented as mean ± SD. The distribution of raw expression values across all ProbeIDs using histograms showed many low-abundance transcripts close to background (noise) levels. These transcripts can result in misleading fold changes and statistically significant results that are not biologically meaningful. Based on this, we set a minimum average raw count of 100. 

### 4.5. MTT Assay

Cells were seeded in 96-well plates. Cell viability was assessed with MTT (3-(4,5-Dimethylthiazol-2-yl)-2,5-Diphenyltetrazolium Bromide; Thermo Fisher Scientific, Waltham, MA, USA). MTT was dissolved to 2 mg/mL in PBS. Then, 50 µL MTT solution was added per well in the cell culture medium after the treatment. After incubating the plate in the dark at 37 °C for two hours, the media were removed and 200 µL DMSO (Roth, Karlsruhe, Germany) was put in every well. The plate was incubated at room temperature for 1 h and every well was mixed. Absorbance was measured at 570 nm (reference 655 nm), and viability was expressed relative to untreated controls.

### 4.6. Immunofluorescence

Cultured cells were fixed, permeabilized, blocked, and incubated with primary antibodies overnight, followed by fluorescent secondary antibodies and DAPI nuclear staining. Images were acquired using automated fluorescence microscopy (ACQUIFER Imaging GmbH, Heidelberg, Germany). Signal intensity was quantified using Fiji/ImageJ (version 2.3.0/1.53f) and normalized to nuclei count as described before [60].

### 4.7. Statistical Analysis

In vitro experiments were performed at least three times independently. Each data from each time point was obtained from different single Transwells. The biological replicates were handled by different batches of cells and technical duplicates by seeding multiple Transwells for each time points and different treatments in parallel. Statistical analyses were conducted using GraphPad Prism 10. Parametric or non-parametric tests were applied as appropriate following normality testing. Data are presented as mean ± SD, or median ± IQR. A *p*-value < 0.05 was considered statistically significant.

## Figures and Tables

**Table 1:** Phosphate transporter genes.

Symbol	EntrezID	Gene Name	Alias
*SLC20A1*	6574	Solute Carrier Family 20 Member 1	*GLVR1*, *Glvr-1*, *PIT1*, *PiT-1*
*SLC20A2*	6575	Solute Carrier Family 20 Member 2	*PiT-2*, *PIT2*, *GLVR2*, *Glvr-2*, *Ram-1*
*SLC34A1*	6569	Solute Carrier Family 34 Member 1	*FRTS2*, *HCINF2*, *NAPI-3*, *NPHLOP1*, *NPT2*
*SLC34A2*	10568	Solute Carrier Family 34 Member 2	*NAPI-3B*, *NAPI-IIb*, *NPTIIb*, *NaPi2b*, *PULAM*
*SLC34A3*	142680	Solute Carrier Family 34 Member 3	*HHRH*, *NPT2C*, *NPTIIc*
*SLC17A1*	6568	Solute Carrier Family 17 Member 1	*NPT1*, *NAPI-1*
*SLC17A2*	10246	Solute Carrier Family 17 Member 2	*NPT3*
*SLC17A3*	10786	Solute Carrier Family 17 Member 3	*GOUT4*, *NPT4*, *UAQTL4*
*SLC17A4*	10050	Solute Carrier Family 17 Member 4	*KIAA2138*
*XPR1*	9213	Xenotropic And Polytropic Retrovirus Receptor 1	*SLC53A1*, *SYG1*, *X3*, *IBGC6*

## Data Availability

The raw data from the RNA-seq from endothelial and mesothelial cell lines were uploaded to ArrayExpress and can be accessed under E-MTAB-12021 [33]. The transcriptomics data of microdissected omental arterioles from children with normal kidney function, with chronic kidney disease and on peritoneal dialysis were generated by our group [35].

## Supplementary Materials

The following supporting information can be downloaded at https://www.mdpi.com/article/10.3390/ijms27083683/s1.

## Abbreviations

The following abbreviations are used in this manuscript:

CKD5	Stage 5 chronic kidney disease
DAPI	4′,6-Diamidin-2-phenylindol
GDP	Glucose degradation product
HCMECs	Human cardiac microvascular endothelial cells
HD	Hemodialysis
HPMCs	Human peritoneal mesothelial cells
HUVECs	Human umbilical vein endothelial cells
IQR	Interquartile range
*K_i_*	Inhibitor constant
LDH	Lactate dehydrogenase
MeT-5A	Immortalized pleural mesothelial cells
MTT	3-(4,5-Dimethylthiazol-2-yl)-2,5-Diphenyltetrazoliumbromid
NHE3	Sodium–hydrogen exchanger 3
NKF	Normal kidney function
P	Phosphate
*P*_app_	Apparent permeability coefficient
PD	Peritoneal dialysis
PFA	Sodium phosphonoformate tribasic hexahydrate
SD	Standard deviation
SEM	Standard error of the mean
TER	Transepithelial electrical resistance
