# Supplementary material for: Analysis of Phosphate Transporters in Peritoneal Cells and Tissues and Their Transport Kinetics In Vitro
Source: Int J Mol Sci. 2026 Apr 21;27(8):3683. doi: 10.3390/ijms27083683 (PMC13115869; doi:10.3390/ijms27083683)
Supplement: Supplementary file 1 [file ijms-27-03683-s001.zip › ijms-4215455-supplementary.pdf]

Supplement data

Patient characteristics

In the omics studies, patients with CKD5, undergoing PD treatment and children with NKF, which is control group, were selected by the same rigorous criteria, such as comparable biochemical findings and PD duration. Children treated by low-GDP used PDF including BicaVera®, Balance® and Physioneal® and high-GDP fluid were Dianeal® and StaySafe®. Biopsies from CKD5 patients were collected during catheter insertion, while samples from control group and PD patients were taken during incidental abdominal surgeries for non-inflammatory issues, catheter exchange, hernia repair, or post-transplant catheter removal. Controls (n = 6), CKD5 (n = 8), low-GDP (n = 5) and high-GDP PD patients (n = 6) were selected for the transcriptome and proteome studies (Table S1).

Table S1. Characteristics of omental arteriolar omics cohorts

Characteristic	Controls (n=6)	CKD5 (n=8)	Low GDP PD (n=5)	High GDP PD (n=6)	p-value
Age [years]	7.6 (7.2)	6.7 (6.1)	9.7 (10.4)	5.4 (6.8)	0.76
Gender [M/F]	5/1	5/3	3/2	3/3	n.a.
BMI	15.7 (3.6)	16.1 (2.5)	17.1 (2.2)	16.6 (5.2)	0.65
PD duration [months]	n.a.	n.a.	21.8 (53.4)	16.0 (27.0)	0.44
GDP exposure [μmol/day/m2 BSA]	n.a.	n.a.	106 (117)	1846 (1286)	<0.01
Hb [g/dL]	12.1 (2.5)	9.7 (3.5)	11.3 (1.3)	10.6 (2.3)	0.04
Ca [mmol/L]	2.4 (1.2)	2.4 (0.5)	2.4 (0.1)	2.4 (0.3)	0.49
P [mmol/L]	n.a.	1.8 (1.1)	1.7 (1.0)	1.5 (0.2)	0.23
PTH [pmol/L]	n.a.	14.2 (67.2)	26.7 (17.0)	14.7 (69.9)	0.79
Creatinine [mg/dL]	0.5 (0.1)	5.6 (3.3)	6.9 (9.3)	6.3 (6.2)	0.02
Albumin [g/L]	47.5 (0.0)	42.1 (15.2)	34.8 (18.6)	33.0 (9.3)	0.29
BUN [mg/dL]	n.a.	79.0 (53.5)	42.9 (34.0)	42.0 (35.7)	0.05

Data are presented as median and interquartile range. Biochemical parameters were measured in serum. GDP = glucose degradation products, PD = peritoneal dialysis, BMI = body mass index, BSA = body surface area, Hb = hemoglobin, Ca = calcium, P = phosphate, PTH = parathyroid hormone, BUN = blood urea nitrogen, n.a. = not applicable.

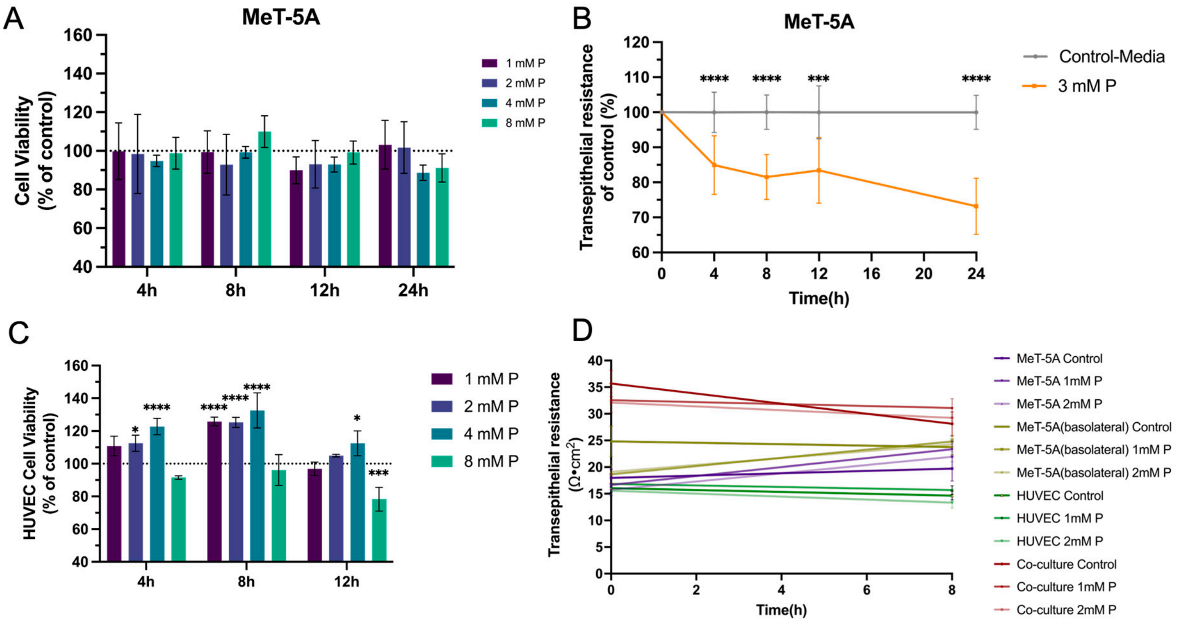

Figure S1. Cell viability of MeT-5A and HUVEC with different phosphate (P) concentrations and transepithelial resistance across 4 different cell layers. **(A)** Cell viability of MeT-5A assessed by MTT assay in media with different concentrations of P over 24 h. No significant toxicity was observed with P concentrations of up to 8 mM. The dashed line represents medium control level. **(B)** Transepithelial resistance of MeT-5A decreased significantly over 24 h when 3 mM P was added in the apical side of Transwell. **(C)** HUVEC were treated with different concentrations of P over 12 h and cell viability was measured by MTT test. 8 mM P induced significant toxicity in HUVEC after 12 h vs. medium control. Lower phosphate concentrations increased the reduction of MTT to Formazan. The dashed line represents medium control level. The increase in phosphate concentrations in the basolateral side of the Transwell with and without HUVEC over time was quantified with 1 mM (Data are mean ± SD. n = 3 experiments in duplicates for A-C). **(D)** Transepithelial resistance across mesothelial and endothelial monolayers and the respective co-culture measured at baseline and after 8 h exposure with 1 mM / 2 mM of P added in the apical side. TER was highest in the co-culture model and lowest in HUVEC.(Data are as mean ± SEM, n =5 with duplicates). * $p < 0.05$, ** $p < 0.01$, *** $p < 0.001$, **** $p < 0.0001$, Two-way ANOVA with Šídák's or Dunnett's multiple comparisons test.

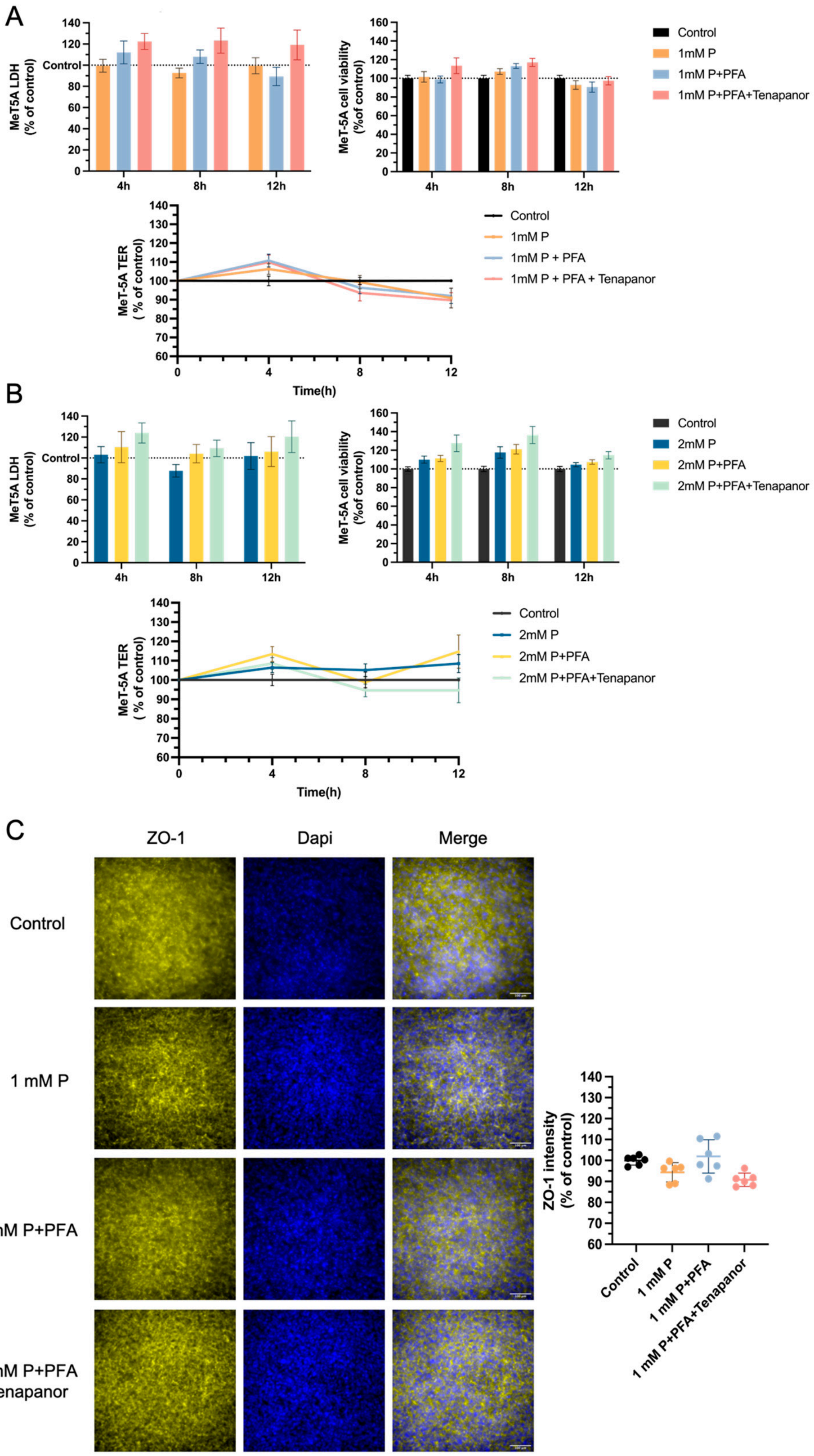

Figure S2. Cell viability and integrity markers of MeT-5A during 12 h of exposure to phosphate and phosphate transport inhibitors. Cell viability was assessed via medium LDH concentrations, by MTT assay and quantification of transepithelial resistance (TER) of MeT-5A during 12 h treatment with 1 mM phosphate (P) (**A**) and 2 mM P (**B**), PFA and Tenapanor. No significant reductions in cell viability, and TER were observed compared to media control. Dashed lines represent medium control level. Data are mean ± SEM. (n = 3 experiments in duplicates. Two-way ANOVA with Tukey'

s and Dunnett's multiple comparisons test were used). (**C**) Representative immunofluorescence stainings of tight junction protein ZO-1 in MeT-5A after 12 h (left images) and digital quantification of ZO-1 abundance (graph on the right side) demonstrate no treatment-depending changes. Transwell filters with MeT-5A were stained and 6 randomly selected areas of each group were imaged by ACQUIFER Imaging Machine with a 20x objective at excitation wavelengths of 385 nm (DAPI), 555 nm (ZO-1 antibody Alexa Fluor 555). The intensity of these areas was quantified with ImageJ. Data are mean ± SD. n = 3 in duplicates. Scale bar = 100 μm.

Figure S3. HUVEC viability and integrity markers of HUVEC and the co-cultures of HUVEC and

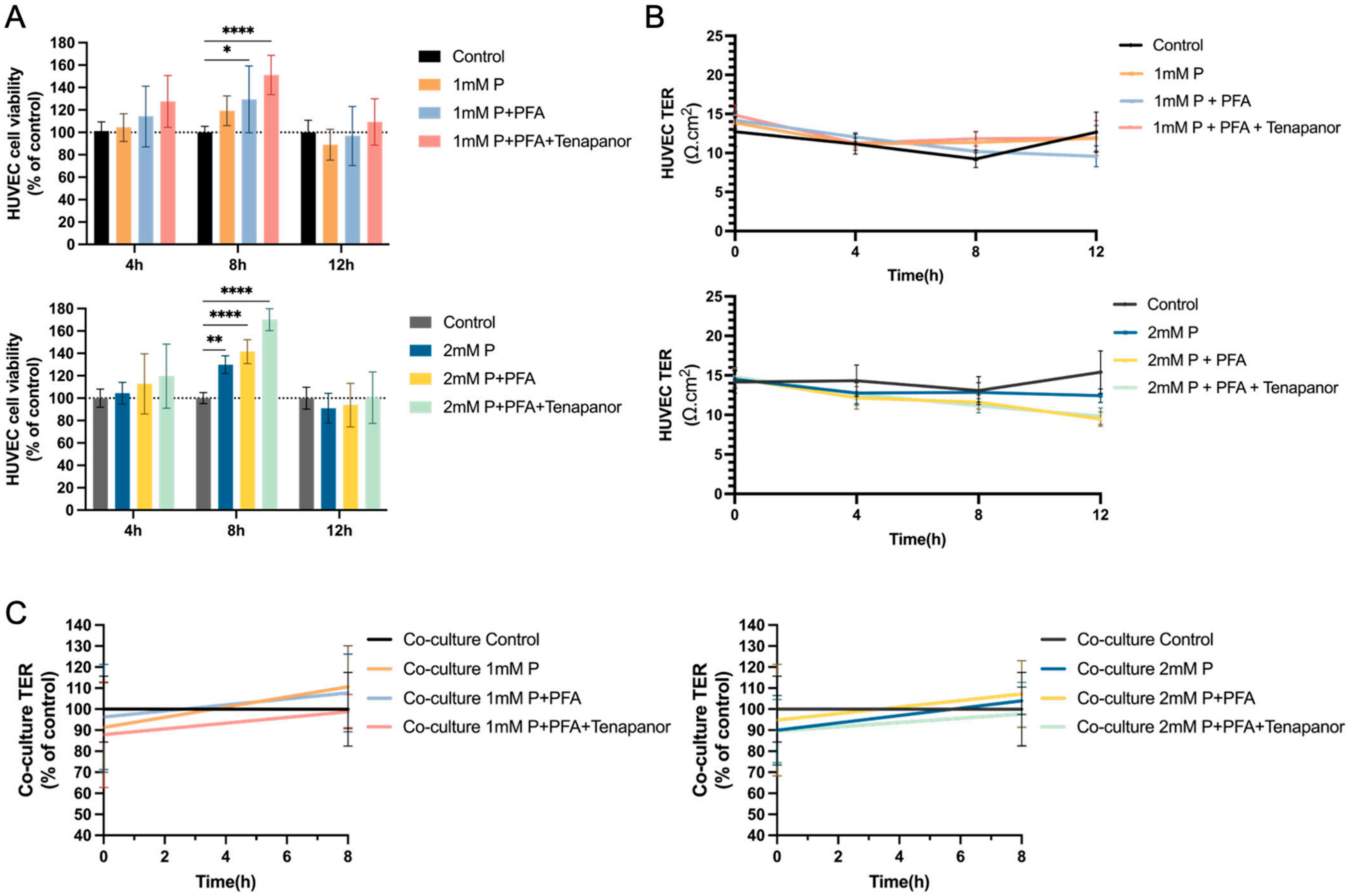

MeT-5A with inhibitors of trans- and paracellular phosphate transport. (**A**) Cell viability of HUVEC was quantified by MTT assay with 1 mM (upper graph) and 2 mM phosphate (P) (lower graph) and inhibitor of transcellular (PFA) and paracellular phosphate transport (Tenapanor). The treatments did not reduce but even increased the reduction of MTT to Formazan (n = 3 experiments with triplicates). Dashed lines represent medium control level. (**B**) Transepithelial resistance (TER) of HUVEC was not affected by these treatments. **(C)** TER of MeT-5A and HUVEC co-cultures before and after 8 h of incubation with phosphate (P) and PFA / Tenapanor treatment was not significantly altered (n = 5 with duplicates). Two-way ANOVA with Dunnett's multiple comparisons was used.
